# Complex Coronary Artery Fistula in a Young Adult: Not Seeing the Wood for the Trees

**DOI:** 10.7759/cureus.49503

**Published:** 2023-11-27

**Authors:** Meriem Boumaaz, Raid Faraj, Zouhair Lakhal, Atif Benyass, Iliyasse Asfalou

**Affiliations:** 1 Department of Cardiology, Mohammed V Military Hospital, Mohammed V University, Rabat, MAR

**Keywords:** case report, non-obstructive coronary arteries, adult congenital heart disease (achd), surgery, cardiac imaging modalities, multidetector computed tomography, coronary artery fistula

## Abstract

Coronary artery fistulas (CAFs) represent rare congenital anomalies that exhibit a wide range of clinical implications and a heightened risk of complications. It is imperative to accurately identify and delineate these fistulas to avoid missed diagnoses and to recommend suitable therapeutic measures.

We present the case of a 46-year-old obese woman who was hospitalized for chest pain associated with palpitations. Clinical examination and ECG results were within normal limits. A 24-hour ECG holter revealed paroxysmal atrial fibrillation. Transthoracic echocardiography revealed a systolodiastolic aliasing originating from the aorta and coursing along the right atrium. Transesophageal echocardiography and coronary angiography confirmed this finding. The diagnosis of a voluminous and tortuous coronary-cameral fistula was established through coronary CT angiography with 3D reconstruction images. A positive stress test indicated the need for surgical closure, given the size and aneurysmal nature of the fistula. However, the patient unfortunately passed away two days after the surgery.

This case highlights the critical need for precise identification and management of CAFs. The patient's unfortunate post-surgical outcome underscores the complexity and risks associated with these anomalies, emphasizing the ongoing need for improved treatment strategies and research in this area.

## Introduction

Coronary artery fistulas (CAFs) are uncommon vascular anomalies that form direct links between a sizable subepicardial coronary artery and either a cardiac chamber or a significant thoracic vessel [1]. While they are typically congenital, acquired forms can also occur [2]. CAFs become clinically significant in adulthood due to an elevated risk of complications, including heart failure, myocardial ischemia, infective endocarditis, arrhythmias, and rupture [3]. The selection of appropriate imaging modalities is extensive. However, it is essential to prioritize techniques that provide a detailed understanding of the origin, trajectory, and entry site of the distal vessel in CAFs, enabling more focused management [4].

In this case report, we present a patient with a voluminous and tortuous coronary-cameral fistula between the right coronary artery and the right atrium, diagnosed using multidetector computed tomography (MDCT) with three-dimensional (3D) volume-rendering images. The primary objective of this paper is to explore various imaging techniques for diagnosing this anomaly, with a specific emphasis on the power of MDCT with 3D volume-rendering images. This approach can assist in making informed decisions regarding the most suitable therapeutic options. The ultimate goal is not only to ensure an accurate diagnosis but also to learn from past therapeutic management decisions, fostering improved clinical outcomes.

## Case presentation

A 46-year-old woman with no significant medical history presented with chest pain that had progressively worsened over the past few months, particularly during physical exertion, and was associated with palpitations. Her only identifiable cardiovascular risk factor was obesity, with a body mass index of 30 kg/m². On initial clinical examination, her heart rate was 75 beats per minute, and her blood pressure measured 130/70 mmHg. The cardiovascular assessment revealed no anomalies, including the absence of heart murmurs or signs of heart failure.

The ECG indicated a regular sinus rhythm at 75 beats per minute without repolarization abnormalities or ventricular hypertrophy. However, a 24-hour ECG holter monitor recorded episodes of paroxysmal atrial fibrillation. BNP levels were assessed and found to be within the normal range.

**Figure 1:**
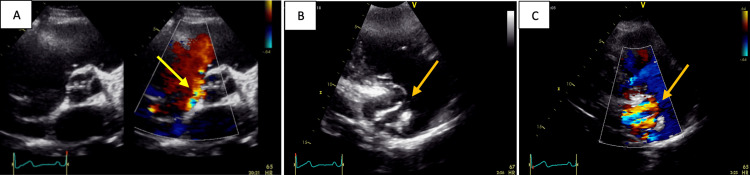
A: Parasternal short axis view showing on color Doppler a systolodiastolic flow originating from the aorta and moving toward the right atrium (yellow arrow). B, C: Modified parasternal long-axis view focused on the right cavities showing a small canal at the wall of the right atrium with aliased flow (orange arrows)

Subsequent transthoracic echocardiography (TTE) demonstrated a non-dilated left ventricle with normal contractile function and a preserved ejection fraction of 65%. In contrast, the right heart cavities were found to be dilated, and the Qp/Qs ratio was estimated at 1:2. Notably, color Doppler imaging revealed a flow pattern with systolodiastolic aliasing that originated from the aorta and coursed along the lateral wall of the right atrium (Figure 1).

**Figure 2:**
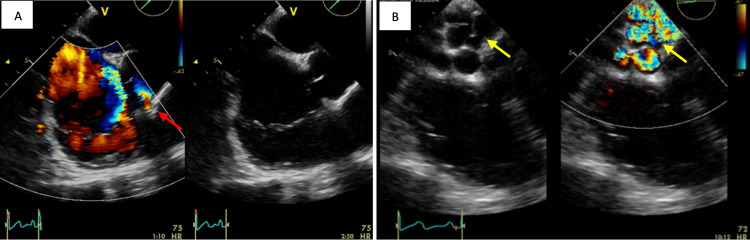
A: Midoesophageal view at 45° showing color Doppler flow originating from the right coronary cusp (red arrow). B: Midoesophageal view at 0° showing several hollow rounded formations filling the right atrium (yellow arrows)

This finding was corroborated through transesophageal echocardiography (TEE), which visualized multiple rounded, hollow structures within the right atrium (Figure 2).

**Figure 3:**
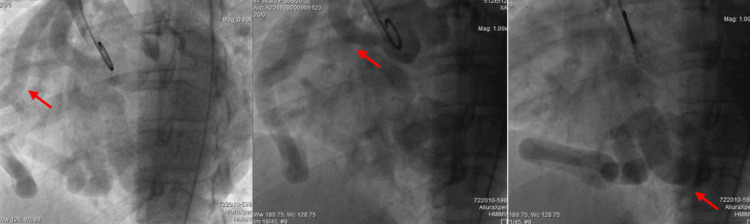
CA showing a healthy ectatic right coronary network (red arrows)

A coronary angiography (CA) demonstrated a dominant and healthy left anterior descending (LAD) artery and a dominant, ectatic right coronary artery (RCA), suggestive of a coronaro-cameral fistula (Figure 3).

**Figure 4:**
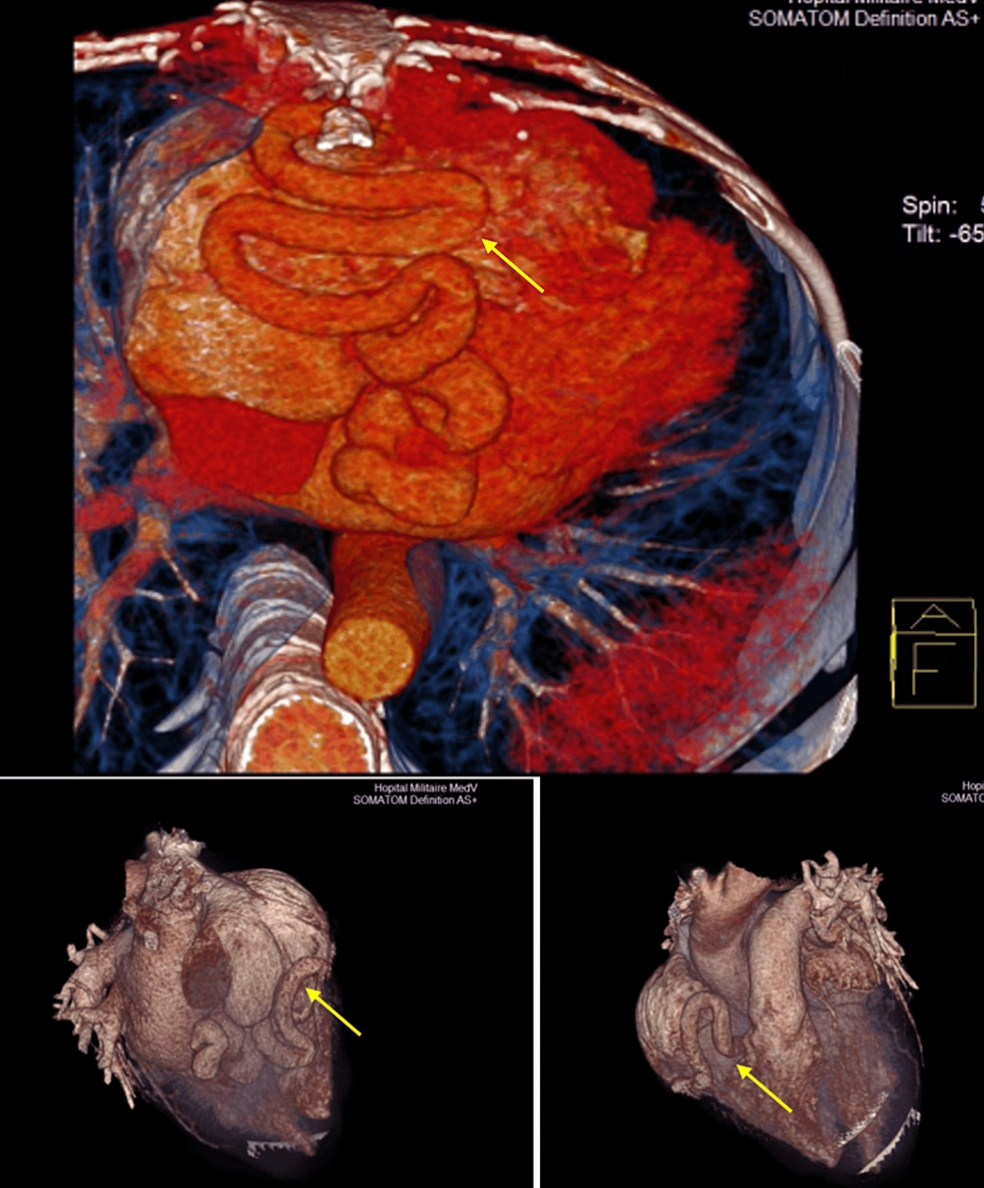
Voluminous and tortuous coronaro-cameral fistula developed between the RCA and right atrium diagnosed by multidetector CT using 3D volume-rendering images (yellow arrows)

Further confirmation was obtained through coronary CT angiography with 3D reconstruction images, definitively confirming the diagnosis of a tortuous and voluminous coronaro-cameral fistula (Figure 4).

**Figure 5:**
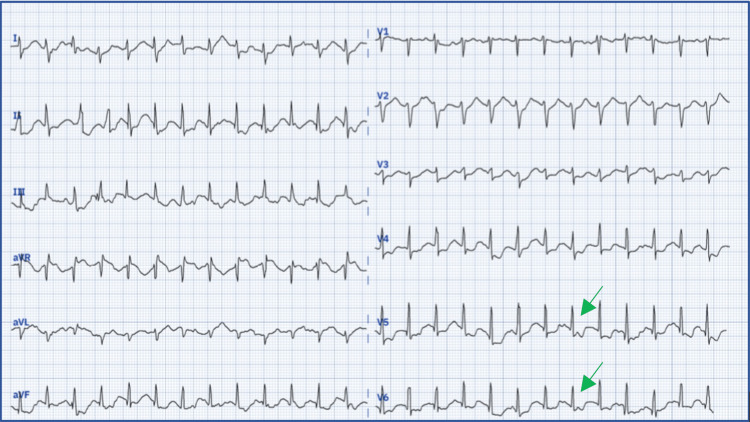
Positive stress test from the second stage with a horizontal ST segment depression of 2 mm in V5 and V6 (green arrows)

A stress test, conducted on a treadmill following the Bruce protocol, produced a positive result, with the development of chest pain and concurrent electrocardiographic changes, specifically horizontal ST segment depression of 2 mm in leads V5 and V6 (Figure 5).

Given the substantial and aneurysmal nature of the fistula, a surgical closure was deemed necessary. During the procedure, a ligature was placed around the coronary artery immediately proximal to the fistula, and the fistula was temporarily occluded. The heart was closely monitored for signs of ischemia, and continuous ECG surveillance was maintained. No signs of ischemia were detected, and myocardial perfusion remained adequate, allowing for the permanent ligation of the fistula. Unfortunately, the patient passed away two days after the procedure due to cardiogenic shock.

## Discussion

CAFs represent uncommon congenital abnormalities. The prevalence of such anomalies is documented at 0.002% in the general population, constituting approximately 0.2% to 0.4% of congenital cardiac anomalies [5]. CAFs are infrequent congenital anomalies, although acquired forms may manifest as complications arising from bypass surgery, coronary angioplasty, and chest trauma [6]. Low-pressure structures are the most common sites of drainage for CAFs, including the pulmonary artery, right-sided chambers, coronary sinus, and superior vena cava [2]. Approximately 50% of CAFs emanate from the RCA. Nevertheless, as per a recent series documented by Tirilomis et al. [7], a preponderance of these anomalies originated from the proximal LAD artery. Congenital fistulas may manifest as isolated anomalies, with infrequent symptomatic occurrences. Alternatively, more commonly, they may be linked with diverse forms of congenital heart disease, marked by either left or right ventricular hypoplasia [4]. A growing body of evidence suggests that most patients develop symptoms as they age, typically in the fifth or sixth decade of life. These symptoms correlate with the gradual, progressive enlargement of the fistula and the augmentation of the shunt [1]. The majority of symptomatic CAFs originate from the RCA rather than the LAD artery [5]. Clinical manifestations comprise angina, dyspnea, arrhythmias, stroke, endocarditis, and myocardial infarction (MI) [1]. The well-recognized relationship between the presence of CAFs and MI results in ischemia through a "coronary steal phenomenon" in the nonexistence of coronary artery disease [8]. The majority of these individuals experience angina during exercise, with myocardial infarction occurring far less frequently [1].

Various imaging methods can be used to assess CAFs. TEE frequently reveals the origin site of congenital CAFs and the distal entry vessel. When CAF is present, it is easy to identify a dilated feeder vessel exhibiting an abnormal flow pattern [2]. Nevertheless, TEE has been associated with various limitations, and a detailed evaluation may be difficult [5]. Multiplane TEE can more accurately define and provide a high-quality panoramic view of the origin, course, and drainage site of CAFs [9]. However, the identification of CAF is constrained when a vessel follows a curvilinear path along the epicardium, hindering the view of distal segments unless dilated; collateral vessels are small and not clearly visualized; and obstructive lesions cannot be precisely assessed [2,9]. CA is effective in determining the size and anatomical characteristics of the fistulous tract but may fall short in illustrating the relationship between CAFs and adjacent structures, as well as the drainage site. Due to its elevated cost and invasive nature, there is growing interest in non-invasive coronary angiography [6]. MRI has been employed in the preoperative assessment of CAFs. Cine MRI sequences offer a detailed representation of flow dynamics, highlighting turbulence at the fistulous communication site, while black-blood sequences provide a clear view of both the vessel lumen and its wall [10]. Nevertheless, MRI comes with inherent drawbacks, such as the requirement for a low heart rate, a regular heartbeat interval (RR interval), and a cooperative patient [10]. MDCT is a fast, noninvasive, and robust method for visualizing the entire coronary artery tree in a single breath-hold [4]. MDCTis significantly faster and offers higher temporal and spatial resolution compared to MRI. Volume-rendered images generated from 3D CT datasets offer an outstanding overview of cardiac and vascular anatomy, aiding interventionists and surgeons in comprehending the anatomical complexity prior to surgery [5,11]. CT can accurately delineate the precise location, shape, and angulation of coronary origins, their anatomical distribution, including dominance, and their trajectory concerning the great vessels. These capabilities can prove particularly beneficial in pediatric cases involving complex congenital diseases [10].

The treatment of CAFs remains a subject of controversy, with recommendations often derived from small retrospective series or anecdotal cases [2]. However, there is a widely shared consensus to address symptomatic CAFs by closing them [1]. Indications for the closure of CAFs encompass a considerable left-to-right shunt, positive findings on a treadmill test, MI, detection of perfusion defects via stress myocardial perfusion imaging, the presence of a mural thrombus, aneurysmal dilation, and the imperative prevention of endoarteritis or rupture [5]. Controversy persists regarding the closure of fistulas in asymptomatic patients. Due to the serious potential long-term complications, it is advisable to treat individuals with moderate to severe shunting [1]. The surgical results for CAFs are deemed satisfactory, establishing surgery as the preferred treatment method. While catheter-based approaches involving various devices, occluders, and coils present acceptable alternatives due to their ease of manipulation, favorable outcomes with high closure rates, and minimal morbidity and mortality rates [12], they may not be suitable for certain patient groups. Instances involving large CAFs with high-flow shunts, multiple communications and terminations, aneurysmal formation, and the need for simultaneous coronary bypass or valve surgery may render transcatheter fistula closure unsuitable for these particular patient cohorts [12]. Complications of surgery include myocardial infarction, arrhythmia, transient ischemic changes, and stroke. Urrutia-S et al. reported only one death in their series. The patient died of congestive heart failure and pulmonary edema on the second postoperative day [13]. Findings from both transcatheter and surgical literature indicate comparable early efficacy, morbidity, and mortality for both methods [2].

## Conclusions

Our case has demonstrated that, in imaging the coronary arteries, MDCT is favored. The unequivocal nature of the high-quality images obtained to define coronary anatomy makes this imaging modality superior to echocardiography or MRI, especially in older children and adults, enabling us to uncover a broader perspective. Although surgical management of CAFs has been successful, our case underscores the potential occurrence of postoperative complications.

## References

[1] Balanescu S, Sangiorgi G, Castelvecchio S, Medda M, Inglese L (2001). Coronary artery fistulas: clinical consequences and methods of closure. A literature review. Ital Heart J.

[2] Gowda RM, Vasavada BC, Khan IA (2006). Coronary artery fistulas: clinical and therapeutic considerations. Int J Cardiol.

[3] Kilic H, Akdemir R, Bicer A, Dogan M (2008). Transcatheter closure of congenital coronary arterial fistulas in adults. Coron Artery Dis.

[4] Doganay S, Bozkurt M, Kantarci M, Erkut B (2009). Coronary artery-pulmonary vein fistula diagnosed by multidetector computed tomography. J Cardiovasc Med (Hagerstown).

[5] Sharma A, Pandey NN, Kumar S (2019). Imaging of coronary artery fistulas by multidetector CT angiography using third generation dual source CT scanner. Clin Imaging.

[6] Gundogdu F, Arslan S, Buyukkaya E, Kantarci M (2007). Coronary artery fistula in a patient with coronary artery disease: evaluation by coronary angiography and multidetector computed tomography. Int J Cardiovasc Imaging.

[7] Tirilomis T, Aleksic I, Busch T, Zenker D, Ruschewski W, Dalichau H (2005). Congenital coronary artery fistulas in adults: surgical treatment and outcome. Int J Cardiol.

[8] Kiuchi K, Nejima J, Kikuchi A, Takayama M, Takano T, Hayakawa H (1999). Left coronary artery-left ventricular fistula with acute myocardial infarction, representing the coronary steal phenomenon: a case report. J Cardiol.

[9] Vitarelli A, De Curtis G, Conde Y (2002). Assessment of congenital coronary artery fistulas by transesophageal color Doppler echocardiography. Am J Med.

[10] Walsh R, Nielsen JC, Ko HH, Sanz J, Srivastava S, Parness IA, Lytrivi ID (2011). Imaging of congenital coronary artery anomalies. Pediatr Radiol.

[11] Schmitt R, Froehner S, Brunn J (2005). Congenital anomalies of the coronary arteries: imaging with contrast-enhanced, multidetector computed tomography. Eur Radiol.

[12] Albeyoglu S, Aldag M, Ciloglu U, Sargin M, Oz TK, Kutlu H, Dagsali S (2017). Coronary arteriovenous fistulas in adult patients: surgical management and outcomes. Braz J Cardiovasc Surg.

[13] Urrutia-S CO, Falaschi G, Ott DA, Cooley DA (1983). Surgical management of 56 patients with congenital coronary artery fistulas. Ann Thorac Surg.

